# Neonatal febrile seizures: Dimethyl itaconate’s role in behavioral recovery and glutathione enzyme modulation in adult rats

**DOI:** 10.1371/journal.pone.0318430

**Published:** 2025-03-25

**Authors:** Zohreh Ghotbeddin, Mohammad Abiri Jahromi, Ali Shahriari, Mohsen Peysokhan, Anahita Memar Dezfouli

**Affiliations:** 1 Department of Basic Sciences, Faculty of Veterinary Medicine, Shahid Chamran University of Ahvaz, Ahvaz, Iran; 2 Stem Cell and Transgenic Technology Research Center, Shahid Chamran University of Ahvaz, Ahvaz, Iran; 3 Department of Veterinary Clinical Sciences, Faculty of Veterinary Medicine, Shahid Chamran University of Ahvaz, Ahvaz, Iran; University of Southern California, UNITED STATES OF AMERICA

## Abstract

Febrile seizures are common in children and can lead to neurological deficits like motor impairments, memory problems, and cognitive decline. Research on dimethyl itaconate aims to mitigate these effects and improve the quality of life for affected people. By exploring its potential as a protective agent against oxidative stress during seizures, this study in adult male rats measures the activity of key enzymes related to oxidative stress and behavioral performance. Pregnant rats were divided into control, sham, DMI, febrile seizure, and DMI +  febrile seizure groups. Seizure severity was evaluated through threshold and frequency measurements, while memory, motor function, and balance were assessed using shuttle box, rotarod, open field, and wire hanging tests. After that, the hippocampus tissue was removed from the brain and the levels of MDA, SOD, GSH, TAC, GR, GPx, and catalase were measured through biochemical methods. Results show that dimethyl itaconate raised the seizure threshold and reduced tonic-clonic seizures. The DMI +  febrile seizure group also showed improved memory, movement, and balance compared to the febrile seizure group (p < 0.05 in all cases). Overall, dimethyl itaconate decreased oxidative stress and improved neurological outcomes in fever-affected rats.

## Introduction

Seizures are sudden and uncontrolled electrical disturbances in the brain that lead to changes in behavior, movements, emotions, and levels of awareness. Having two or more seizures at least 24 hours apart is considered epilepsy. Animal models of neonatal seizures are suitable for studying epilepsy, its mechanisms, and the development of appropriate antiepileptic drugs [1]. Typically, two animal models are used to induce neonatal seizures: hypoxia-ischemia, and fever. It has been reported that 16% to 56% of infants who experience these types of seizures develop epilepsy later in life [2]. By inducing a temperature above 38 degrees Celsius (fever), simple seizures occur first, followed by prolonged seizures (lasting more than 15 minutes), which create a predisposition for temporal lobe epilepsy (TLE) [1]. One of the complications of febrile seizures in infancy is behavioral disorders at later ages. A study by Yagoubi et al. in 2015 showed that rats exposed to hyperthermia for about 30 minutes experienced generalized seizures and impairments in learning and memory in the Morris water maze test [3].

Oxidative stress is recognized as a potential factor in the development of epilepsy [4]. Furthermore, persistent seizures lead to cellular harm by inducing heightened oxidative stress [5]. Several animal models of epilepsy have shown a notable rise in reactive oxygen species (ROS) following seizures, along with major alterations in the antioxidant system of both animals and epileptic people [6].

The sensitivity of the developing brain to oxidative stress is high because it has the highest oxygen consumption, a high level of unsaturated fatty acids that are susceptible to lipid peroxidation, elevated iron levels that can catalyze and form hydroxyl free radicals, and low amounts of catalase (CAT), which increases the brain’s vulnerability to damage caused by oxidative stress [5].

Itaconate is a chemical mostly synthesized by immune cells, particularly macrophages. It has been discovered that itaconate is essential for activating the anti-inflammatory factor Nrf2 by LPS [7]. Dimethyl itaconate (DMI) is a cell-permeable derivative of itaconite [8]. DMI is widely recognized for its ability to stimulate the activation of Nrf2 and enhance the production of Nrf2 protein [7]. NRF2 is a crucial transcription factor involved in the regulation of inflammatory responses and oxidative stress. Activation of NRF2 enhances the activity of enzymes such as HO-1 and controls the formation of glutathione (GSH), resulting in protective benefits against oxidative stress [9]. Multiple proteins are stimulated by NRF2 to protect against cytotoxicity, oxidative stress, and cell demise. Itaconate exhibited a protective effect in liver cells and decreased oxidative stress by activating NRF2 signalling; these results suggest that itaconate possesses antioxidant properties in non-immune cells [10]. In doxorubicin-induced cardiotoxicity, DMI decreased ROS and malondialdehyde levels and inhibited oxidative stress via the activation of the HO-1/NRF2 pathway [11].

Regarding the role of oxidative stress in the pathophysiology of seizures, this research aims to explore how dimethyl itaconate may have a potential therapeutic avenue for protecting neuronal health during seizure events by measuring the activity of glutathione-dependent enzymes and behavioral performance in adult rats affected by seizures.

## Materials and methods

### Animals and grouping

The current research project received approval from the Institutional Ethics Committee of Shahid Chamran University of Ahvaz and followed the NIH guidelines for laboratory animal care. The animals kept under standard conditions, including 50% humidity and a temperature range of 23 ±  2°C, with a 12-hour light/dark cycle. Every animal was allowed to freely eat and drink.

Five experimental groups were created with ten male newborn rats in each group including: A control group that receives no unique treatment. The sham group was given sunflower oil (DMI solvent) on the 10th day after being born. Group of rats experiencing febrile seizures: On the 10th day after giving birth, rats in this group were placed in a fever box for 30 minutes. DMI group: The rat was administered a single dose of 20 mg/kg of DMI on the ninth day postnatal. DMI and febrile seizure group: the animals of this group received DMI at a dose of 20 mg/kg 24 hours before applying fever. All injections were done intraperitoneally.

### Fever induction

Within 10 days after birth, the newborns were moved into a plexiglass box. This container had air intake and exhaust valves installed. The core temperature of approximately 41°C (similar to a serious fever) was reached by using a controlled flow of relatively warm air to increase the temperature. A thermometer was used to monitor the core temperature consistently every 2 minutes. A period of 30 minutes was intentionally used to induce hyperthermia. The intensity of seizures due to fever in each animal was evaluated by measuring two factors: seizure threshold and the frequency of tonic-clonic seizures [12].

### Behavioral tests

Behavioral assessments were conducted from PND45 to PND49. The open field, RotaRod, and wire hanging tests were employed to assess balance and locomotor activity, while the shuttle box test was utilized to evaluate passive memory.

#### Open field test.

The assessment involved tracking animal movement patterns in a 50 × 50 × 50 cm cube box with a camera. Rats were given time to adjust in the lab before being placed in the box. Their movements were recorded for 5 minutes, measuring distance traveled, speed, rearing, and grooming. Testing was done between 9:00 am and 2:00 pm, aligning with the animal’s active hours. Behavioral tests are most effective in the morning to early afternoon. [13].

#### Rotarod test.

The Rotarod test assesses balance and coordination in rodents using a rotating wheel at varying speeds. Rats were acclimated for two training sessions on the rotating rod. Their ability to maintain balance was tested three times, each lasting 5 minutes with a 1-2 minute interval. The average performance duration was recorded in seconds. The instrument was sterilized with 70% alcohol after each test, with consistent laboratory conditions for all animals [14].

#### Wire hanging tests.

This device is used to assess motor coordination, examine deficiencies in the sensory-motor system, and evaluate balance in rodents. The mesh is square or circular, with a diameter of approximately 40 centimeters, consisting of mesh squares with a diameter of 1 centimeter, surrounded by wood with a diameter of 4 centimeters. For this purpose, rats were placed in the center of a rectangular box (60 centimeters long and 50 centimeters wide), which was then immediately inverted and held 50 centimeters above the ground. The duration of the rat’s fall from the box was noted. The experiment was repeated three times for every rat, and the final result was the average time (in seconds) of these repetitions [15].

#### Shuttle box test.

The shuttle box device has two chambers, one light and the other dark, it consists of three stages: habituation, training, and testing. In the habituation phase, animals are familiarized with the device without any shock by spending time in the bright chamber, then given the opportunity to move to the dark chamber within 60 seconds. Failure to do so indicates poor motivation. During the training phase, animals are shocked in the dark chamber after a delay. In the retrieval or testing phase, the rat is placed in the illuminated area after 24 hours for recall and long-term memory evaluation. The latency time of time before entering the dark chamber and the duration spent inside were recorded. The longest time to spend in either the dark or light areas is 300 seconds [16].

### Evaluation the oxidative stress parameters

**Fig 1:**
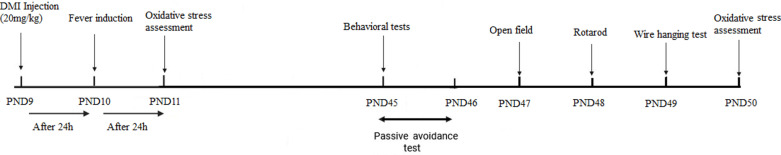
The timeline of the experiment.

After behavioral test, the animals sacrificed with carbon dioxide Inhalation which is set with the guidelines by Shahid Chamran Veterinary Medicine and accordance with the National Institutes of Health (NIH).Oxidative stress was assessed by measuring the activity of glutathione-dependent enzymes such as MDA, SOD, GSH, TAC, GR, GPx, and catalase from the hippocampus tissue of rats in two time periods immediately after fever and at puberty after completing behavioral tests. The time line of experimental procedure was shown at Fig 1.

#### Measurement of reduced glutathione (GSH).

The study used Ellman’s method to measure reduced glutathione levels by reacting a reagent with sulfidyl groups to produce a colored complex. A virtual blank was created with a buffer and reagent solution, and samples were mixed with a buffer solution and ethylenediaminetetraacetic acid. After adding an element reagent, the mixture was incubated and absorption was measured at 412 nm using a microplate reader. The goal was to quantify glutathione levels through optical absorption [17].

#### Measurement of catalase (CAT).

This study measured catalase enzyme activity using a method developed by Koroluk et al. in 1988. The test involves the reaction of hydrogen peroxide with ammonium molybdate to produce a yellow compound, which is quantified at 410 nm using spectrophotometry. Catalase in the sample inhibits this reaction by breaking down hydrogen peroxide. An international unit (IU) of enzyme is the amount needed to decompose one micromol of hydrogen peroxide into water and oxygen in a minute. The process includes adding Tris HCl buffer and hydrogen peroxide to wells, followed by ammonium molybdate after 10 minutes. Absorbance at 410 nm is measured, and catalase concentration is calculated using a formula involving optical absorbance values and control concentration [17].

#### Measurement of superoxide dismutase (SOD) activity.

Superoxide dismutase enzyme activity can be quantified using photometric methods with a specific absorption wavelength. These methods involve generating superoxide radicals and measuring their reaction with the I.N.T. chromogen to produce a red formazan color. Inhibition of this reaction allows for measuring SOD enzyme activity. Assessment was done at 505 nm using a microplate reader at 0 and 3 minutes. One unit of SOD inhibits 50% of the color generation reaction. The enzyme concentration is expressed in international units per gram of protein [18].

#### Malondialdehyde (MDA) activity measurement.

The quantity of malondialdehyde (MDA) in a sample was determined by combining 100 microliters of the sample with 200 microliters of a working solution in microtubes, which were then boiled in water for 15 minutes. The samples were then agitated and cooled, with their optical absorption measured at 535 nm. The MDA quantity was calculated using the equation C =  OD/1.56 ×  105, and results were expressed in micromoles per liter. This method, based on Pelser et al.‘s 1996 methodology with modifications, involved heating the sample with thiobarbituric acid (TBA) to form a purple complex whose intensity correlates with MDA quantity, measured at 532 nm with a spectrophotometer [19].

#### Measurement of total antioxidant capacity.

This test assesses the liver tissue’s ability to convert Fe3 + -TPTZ to Fe2 + -TPTZ using a pH of 3.6 and a temperature of 25°C, resulting in a blue color measured by spectrophotometry. Calculations are done with a standard curve using FeSO4.7H2O. An optical absorption test is conducted by creating a virtual blank of distilled water and a working solution. Antioxidant activity in blood plasma is standardized with various concentrations. Samples are mixed with a working solution containing acetate buffer, and TPTZ, then incubated at 37°C for 10 minutes. The mixture is analyzed at 593 nm using a micro plate reader with a blank present [20].

### Statistical analysis

The results were reported as the mean ±  the standard error of mean (Mean ±  SEM). The statistical analyses and graphs were performed using the GraphPad Prism 9 statistical software, with a significance level set at p < 0.05. The statistical comparison of various parameters among the experimental groups was conducted using a one-way ANOVA and Tukey’s post hoc test. Seizure activity results between febrile group and DMI + febrile seizure group was analyzed using an Unpaired T-test and Two-way ANOVA was used to compare oxidative stress between two period of time at P12 and P45.

## Results

The findings from this research are categorized into four sections investigating the effect of dimethyl itaconate on 1) seizures, 2) movement and coordination, 3) memory, and 4) oxidative stress.

### Seizure activity results

**Fig 2:**
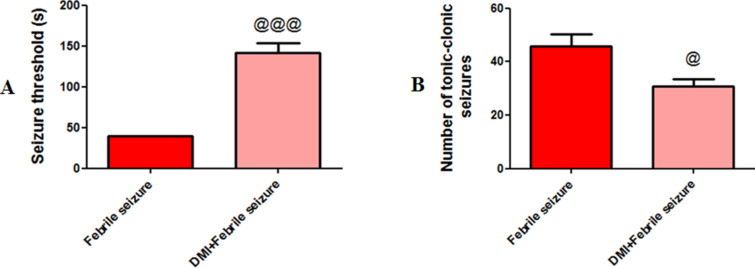
Comparison the average of A) seizure threshold and B) the number of tonic-clonic seizures in febrile and DMI +  febrile groups. @ (p < 0.05) and @@@ (p < 0.001) indicates a significant difference between the DMI+Febrile group compared to the febrile group. Data are presented as mean ±  SEM (n = 10).

The study examined the impact of dimethyl itaconate on convulsive activity in 10-day-old newborns by measuring seizure threshold and number of tonic-clonic seizures. Results showed that the group injected with dimethyl itaconate had a significantly higher seizure threshold 142 ±  11.72 (p <  0.001) (Fig 2A) and lower number of seizures 30.66 ±  2.84 (p < 0.05) (Fig 2B) compared to the fever-only group (39.33 ±  0.66, 45.67 ±  4.70). These findings were analyzed using an Unpaired T-test, demonstrating the beneficial effects of dimethyl itaconate on reducing convulsive activity in newborns.

### Movement, balance and coordination results

This section examines the total movement, speed of movement, and the number of rearing (raising on two legs) as measures of motor activity in the open field test. The study examined and compared the duration of maintaining balance on the RotaRod revolving rod and the duration of maintaining balance on the wire hanging test to evaluate balance.

#### Open field results.

**Fig 3:**
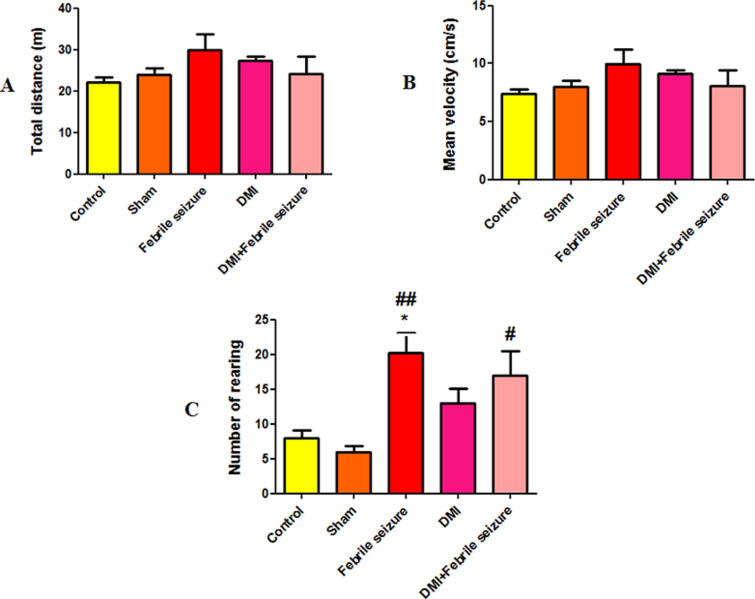
Comparison the average of A) total distance and B) mean velocity and C) number of rearing between groups. *  (p < 0.05) indicates a significant difference compared to the control group. # (p < 0.05) and ## (p < 0.01) show a significant difference compared to the sham group. Data are presented as mean ±  SEM (n = 10).

According to the results obtained from the open field test, no significant difference was observed between the studied groups in the average of total traveled distance and movement speed (Fig 3A and 3B). The results of the average frequency of rearing show a significant increase in the febrile group 20.2 ±  compared to the control (p <  0.05) and the sham group (p <  0.01). It was also found that in the DMI + febrile group, the number of rearing increased significantly compared to the sham group (p < 0.05) (Fig 3C).

#### The results of balance maintenance on the rotarod.

**Fig 4:**
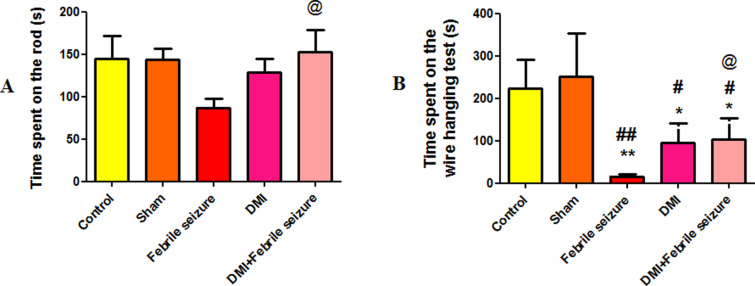
The average of time spent on the A) rotarod and on the B) wire hanging test. *  (p < 0.05) and ** (p < 0.01) show a significant difference compared to the control group. # (p < 0.05) and ## (p < 0.01) present a significant difference compared to the sham group and symbol @ indicates a significant difference between the DMI+febrile seizure and febrile seizure groups. Data are presented as mean ±  SEM (n = 10).

The results indicate a significant increase in the average time of staying on the rotating rod and maintaining balance in the febrile seizure group which are pretreated with DMI, compared to the febrile group (p < 0.05) (Fig 4A).

#### The results of the balance maitanace in the wire hanging test.

Significant decreases in balance duration were seen in the febrile group compared to control and sham groups (p < 0.01). DMI alone and DMI+febrile seizure groups also showed reduced balance times compared to control and sham groups (p < 0.05). DMI injection 24 hours before fever onset increased balance duration in the febrile group (Fig 4B).

### Memory results

The shuttle box test was used to measure passive avoidance memory.

#### Shuttle box test result.

**Fig 5:**
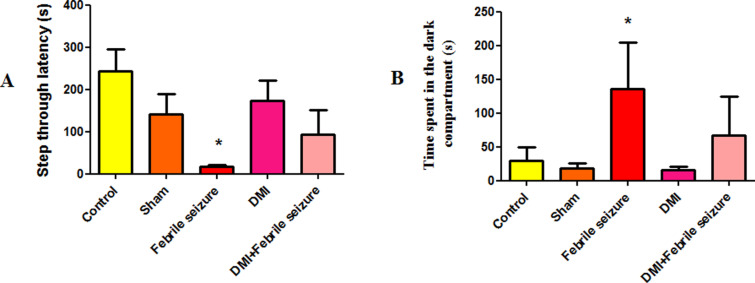
The average of A) delay time to enter in to the dark room (in seconds) and B) time spent in the dark compartment in the shuttle box test. *  indicates a significant difference between the febrile group and the control group at the level of (p < 0.05). Data are presented as mean ±  SEM (n = 10).

Comparing the delay time to enter in to the dark part of the shuttle box showed that the febrile group entered more quickly than the control group (p < 0.05) (Fig 5A). On the other hand, the febrile group spent significantly more time in the dark section of the box than the control group, with a statistically significant difference (p < 0.05) (Fig 5B).

### Results of oxidative stress and activity of glutathione-dependent enzymes

Oxidative stress in rat hippocampal tissue was measured by assessing glutathione-dependent enzymes (MDA, SOD, GSH, TAC, GR, GPx, and catalase) after fever and during adolescence post-behavioral tests. Diagrams compared immediate (P12) and later (P45) oxidative stress levels between experimental groups using One-way ANOVA and Tukey’s post hoc test. Additionally, Two-way ANOVA compared oxidative stress levels at P12 and P45.

#### Malondialdehyde (MDA).

**Fig 6:**
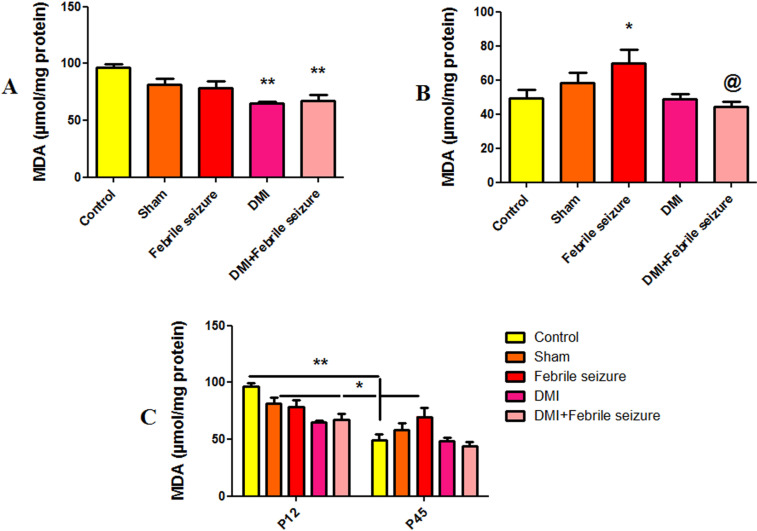
Comparison of the average activity of MDA. * (p <  0.05) and ** (p <  0.01) show the difference compared to the control group and @ (p <  0.05) show the difference between the DMI+febrile group and the febrile group. Part A of each graph shows the difference between experimental groups in 12-day-old rat (immediately after fever), part B shows the difference between experimental groups in 45-day-old rat (after the end of behavioral tests), and part C shows the difference between each group in two time periods (12 and 45 days). Data are shown as mean ±  standard error.

The study used the MDA index to measure lipid peroxidation levels and found a significant decrease at P12 in MDA activity in both the DMI and DMI +  febrile groups compared to the control group (p < 0.01) (Fig 6A). In the 45-day-old rat, the febrile group showed an increase in MDA levels compared to the control (p < 0.05) but a decrease when combined with DMI (p < 0.05) (Fig 6B). There was also a reduction in MDA activity in 45-day-old rat compared to 12-day-old in both control and experimental groups, indicating a decrease in MDA activity over time. The enzyme function was significantly reduced in both groups (Fig 6C).

#### Superoxide dismutase (SOD).

**Fig 7:**
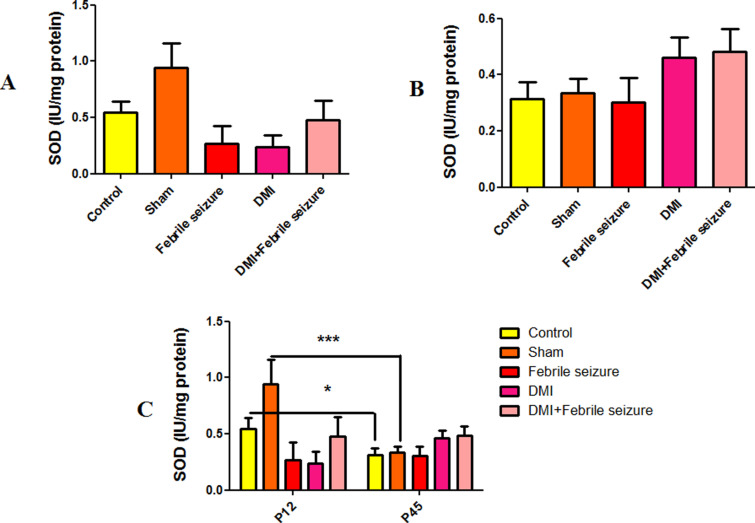
Comparison of the average level of SOD activity. *  (p <  0.05) and *** (p <  0.001) show the difference between the control and sham groups in two periods of 45 and 12 days. Data are shown as mean ±  standard error.

Superoxide Dismutase levels showed no significant difference between groups at 12 and 45 days, as displayed in Fig 7 (part A and B). However, in part C, a significant difference was observed between the 45-day-old rat and the 12-day-old rat of the control and sham groups, and the activity in the 45-day-old groups was significantly reduced compared to the 12-day-old with statistical significance at p < 0.05 and p < 0.001, respectively (Fig 7C).

#### Catalase.

**Fig 8:**
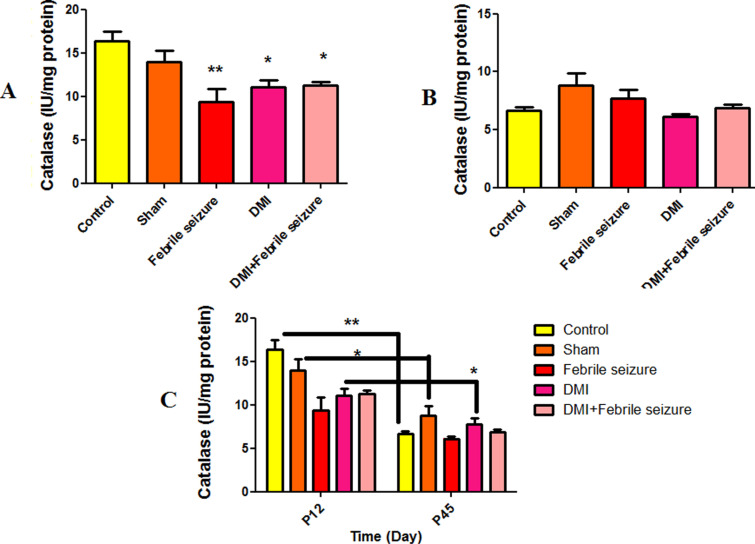
Comparison of the average level of catalase activity. In part A of the diagram, *  (p <  0.05) and ** (p <  0.01) show the difference compared to the control group. In part C, *  (p < 0.05) and ** (p < 0.01) show the difference between the control, sham and DMI groups of 45-day and 12-day-old rat. Data are shown as mean ±  standard error.

Analyzing catalase activity in different experimental groups, the 12-day-old rat in part A exhibited notable decreases in groups with fever (P < 0.01), fever with DMI, and DMI alone (P < 0.05) compared to the control (Fig 8A). Catalase activity showed a significant difference between control (P < 0.01), sham (P < 0.05) and DMI group (P < 0.05) of 45-day and 12-day-old rat (Fig 8C).

#### Reduced glutathione (GSH).

**Fig 9:**
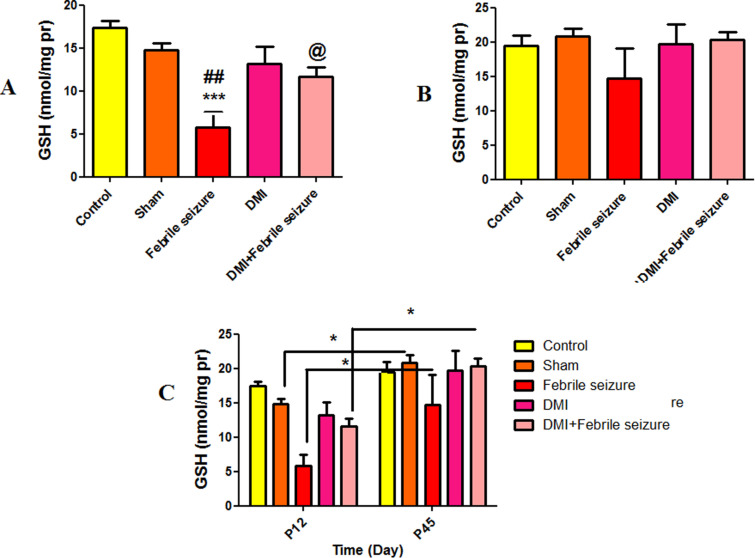
Comparison of the average level of reduced glutathione. In part A: *** indicates a significant difference compared to the control group (p < 0.001), ## (p < 0.01) compared to the sham group, and @ shows the difference between the DMI + febrile group and the febrile group. part C: There was a significant difference between the sham, DMI, and DMI + febrile groups of 45-day-old and 12-day-old rat (p < 0.05). The data are presented as mean ±  standard error.

Fig 9A shows a significant decrease in reduced glutathione levels in the febrile group compared to control (p < 0.001) and sham groups (p < 0.01). The DMI+febrile group exhibited a significant increase in reduced glutathione levels compared to the febrile group (p < 0.05). In 45-day-old rat, there was no significant difference in reduced glutathione levels among the experimental groups (as indicated in Fig 9B). By examining Fig 9C of the chart, we notice that the level of reduced glutathione in the control, febrile, and DMI + febrile groups of 45-day-old rat has significantly increased compared to 12-day-old rat (p < 0.05).

#### Total antioxidant capacity (TAC).

**Fig 10:**
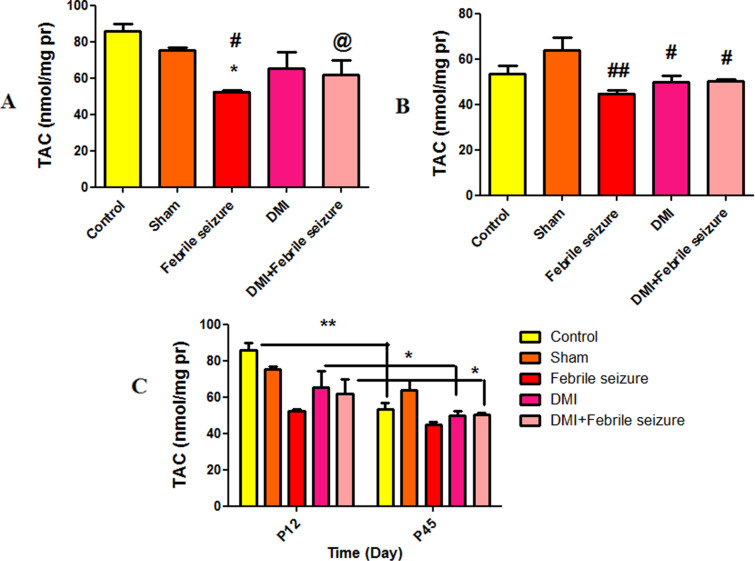
Comparison the total of antioxidant capacity (TAC) mean. In part A and B: # (p < 0.05) and ## (p < 0.01) indicate a significant difference compared to the sham group, *  indicates a significant difference compared to the control group (p < 0.05), and @ show a significant difference between the febrile group with dimethyl itaconate injection and the febrile group alone (p < 0.05). part C: *  (p < 0.05) and ** (p < 0.01) show the difference between the control group, DMI group, and the DMI + febrile group of 45-day-old rat compared to the 12-day-old rat.

The results obtained from measuring the total antioxidant capacity in Fig 10A indicate that its level in the febrile group significantly decreased compared to the control and sham groups (p < 0.05). Meanwhile, the total antioxidant capacity in the DMI+febrile group showed a significant increase compared to the febrile group (p < 0.05). In 45-day-old rat, a significant difference was also observed between the febrile (p < 0.01), DMI (p < 0.05), and DMI + febrile groups (p < 0.05) compared to the control group (Fig 10B). Over time, there was a significant difference between the control groups (p < 0.01), DMI (p < 0.05), and the DMI+febrile group (p < 0.05) of 45-day-old rat compared to 12-day-old rat (Fig 10C).

#### Results of glutathione peroxidase (GPx).

**Fig 11:**
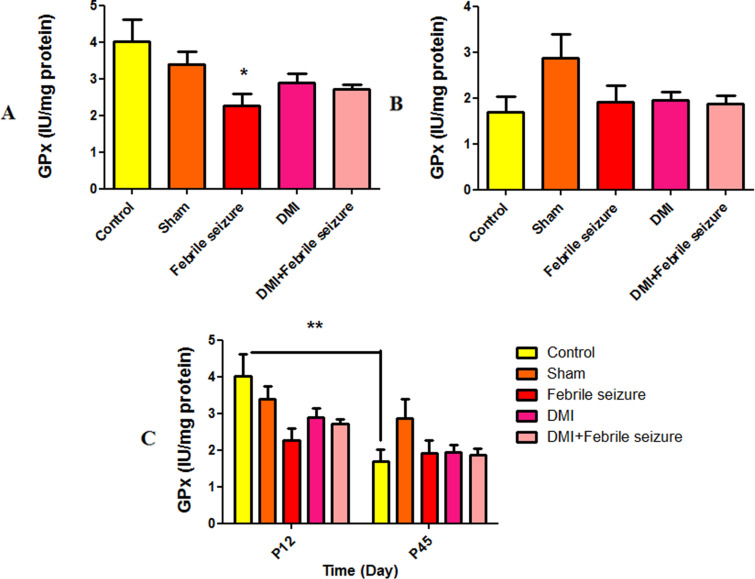
Comparison the mean of glutathione peroxidase. Part A: *  Indicates a significant difference between the febrile group and the control group (p < 0.05). Part C: *  Shows the difference between the groups at two time intervals of 45 and 12 days. The 45-day control group showed a significant difference compared to the 12-day group (p < 0.01). The data are presented as mean ±  standard error.

The analysis of the results at Fig 11A shows that glutathione peroxidase levels in the febrile group were lower compared to the control group, with a significant difference at (p < 0.05). No significant difference was observed between the experimental groups in Fig 11B. The GPx in the control group of 45-day-old rat showed a significant decrease compared to the 12-day-old rat (Fig 11C) (p < 0.01).

#### Glutathione reductase (GR) results.

**Fig 12:**
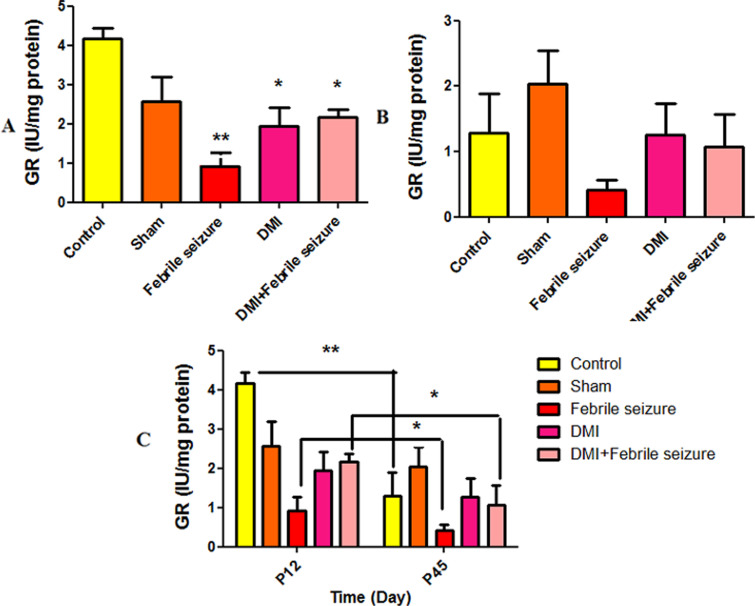
Comparison of average glutathione reductase. Part A: A significant difference was observed between the febrile group (p < 0.01), DMI group (p < 0.05), and the DMI + febrile group (p < 0.05) compared to the control group. Part C: *  Shows the difference between the groups at two time intervals of 45 and 12 days. Glutathione reductase in the control group (p < 0.01), febrile group (p < 0.05), and DMI + febrile group (p < 0.05) showed a significant decrease at 45 days compared to 12 days. The data are presented as mean ±  standard error.

The results indicate that glutathione peroxidase significantly decreased in the three groups: DMI (p < 0.05), febrile (p < 0.01), and febrile combined with DMI (p < 0.05) compared to the control group (Fig 12A). No significant differences were observed among the studied groups at 45 days (Fig 12B). Glutathione reductase showed a significant decrease in the control group (p < 0.01), febrile group (p < 0.05), and the DMI +  febrile group (p < 0.05) on day 45 compared to day 12 (Fig 12C).

## Discussion

This study utilized hyperthermia to induce febrile seizures in newborn rats between (PND10), a critical period for synaptic development. Fever is a common trigger for seizures in infants, sometimes leading to temporal lobe epilepsy later on. Researching febrile seizures presents multiple benefits compared to another seizures models like hypoxia-induced neonatal seizures because of their common occurrence, consistent triggers, and reduced risk of lasting injury, established treatment strategies, and the possibility for genetic discoveries. Inducing fever through hyperthermia decreased seizure threshold and increased tonic-clonic seizures compared to a control group. Research has shown a 30-minute temperature increase, can trigger seizures. Hyperthermic seizures in 10-11 day old rats led to structural changes in the hippocampus and amygdala, with no significant neuronal death observed [21]. These seizures increased presynaptic inhibitory transmission in the hippocampus, altering excitation/inhibition balance in neural circuits [22]. These changes persisted into adulthood, indicating a shift in GABAergic system activity. Studying febrile seizures in animal models provides insights into their causes and impacts, paving the way for future research and treatments [23]. The use of newborn rats at this specific age corresponds to the developmental stage of human infants [24].

The findings indicating that dimethyl itaconate (DMI) raises seizure threshold and reduces the occurrence of tonic-clonic seizures present a compelling avenue for further exploration in the context of epilepsy management. DMI may offer neuroprotective benefits, potentially through intricate mechanisms that require further study. As a metabolic modulator, DMI influences biochemical pathways, being a derivative of itaconate with anti-inflammatory properties. In epilepsy, CNS inflammation can exacerbate seizures [11]. DMI may stabilize neuronal activity by modulating inflammation, thus increasing seizure threshold [25]. Itaconate and its derivatives impact metabolic processes like the TCA cycle and oxidative stress responses. DMI’s potential to enhance mitochondrial function and decrease oxidative stress could safeguard neurons from excitotoxicity, crucial for preventing neurodegeneration in chronic seizure disorders [26].

The study found that seizures induced by hyperthermia resulted in behavioral disturbances such as reduced memory and balance compared to the control group, aligning with existing literature emphasizing the detrimental effects of early-life seizures on long-term neurodevelopment. Hyperthermia is a known trigger for seizures, particularly in pediatric populations, with the developing brain being particularly vulnerable to the effects of elevated temperatures [27]. Seizures during this critical developmental window can lead to lasting cognitive impairments due to alterations in neuronal connectivity and synaptic plasticity [28].

Memory deficits may be linked to hippocampal damage caused by seizures, while balance disturbances could be a result of cerebellar dysfunction [29]. Understanding how hyperthermia impacts brain development could lead to targeted therapies aimed at preventing or reducing the impact of seizures on neurodevelopment.

DMI shows promise in improving memory and balance deficits caused by early postnatal febrile seizures. Research indicates that DMI, an electrophilic metabolite, can modulate inflammation and oxidative stress, both critical factors in seizure-related impairments [30]. It also affects crucial neuronal metabolic pathways and inhibits NLRP3 inflammasome activation, decreasing neuroinflammation [31]. These actions may support neurogenesis and synaptic plasticity, vital for memory and motor coordination. Additionally, DMI’s role in reducing neuroinflammation could benefit cerebellar neurons, potentially restoring balance and coordination.

Our findings indicate that febrile seizures significantly increase oxidative stress, while DMI effectively mitigates this oxidative burden. This reduction in oxidative stress may underlie the observed improvements in behavioral outcomes, including enhanced memory and motor coordination.

Oxidative stress, characterized by an imbalance between reactive oxygen species (ROS) production and antioxidant defenses, is a well-documented consequence of seizure activity [32]. Increased ROS can lead to neuronal damage, inflammation, and ultimately contribute to cognitive deficits [33].

In the context of febrile seizures, oxidative stress can exacerbate neuronal injury, particularly in vulnerable brain regions such as the hippocampus and cerebellum [34]. Our results align with previous studies that have shown elevated levels of oxidative stress markers following seizures, further supporting the hypothesis that oxidative damage plays a pivotal role in seizure-related neurodevelopmental impairments [35].

DMI is known for its electrophilic properties that allow it to modify cysteine residues on proteins, thereby influencing various signaling pathways related to inflammation and oxidative stress [36].mBy reducing oxidative stress, DMI may help restore the balance of redox homeostasis in the brain, which is crucial for maintaining neuronal health and function. Our findings demonstrate that DMI administration significantly reduces oxidative stress levels following febrile seizures. This effect could be attributed to DMI’s ability to enhance glutathione-dependent enzyme activity, which plays a critical role in detoxifying ROS. Glutathione peroxidase and glutathione reductase are key enzymes in the glutathione pathway that help mitigate oxidative damage [37].

By upregulating these enzymes, DMI may facilitate a more robust antioxidant response, thus protecting neurons from oxidative injury. Previous research has established a strong link between oxidative stress and cognitive deficits; excessive ROS can impair synaptic plasticity, a fundamental process underlying memory formation [38]. Furthermore, oxidative damage to cerebellar neurons can lead to motor coordination issues [39].

By alleviating oxidative stress through DMI treatment, we may be observing a restoration of both hippocampal and cerebellar function.

## Conclusion

The findings from this study suggest that DMI reduce oxidative stress and could serve as a promising therapeutic agent for mitigating the long-term consequences of febrile seizures in early life. Given that traditional antiepileptic drugs often focus on seizure control without addressing underlying neurodevelopmental issues, the use of metabolic modulators like DMI may offer a novel approach to treatment.

## Supporting information

S1 Data Row data. (RAR)

S1 File Graphical abstract. (JPG)
