# Supplementary figures and images for: Neonatal febrile seizures: Dimethyl itaconate’s role in behavioral recovery and glutathione enzyme modulation in adult rats
Source: PLoS One. 2025 Mar 25;20(3):e0318430. doi: 10.1371/journal.pone.0318430 (PMC11936269; doi:10.1371/journal.pone.0318430)

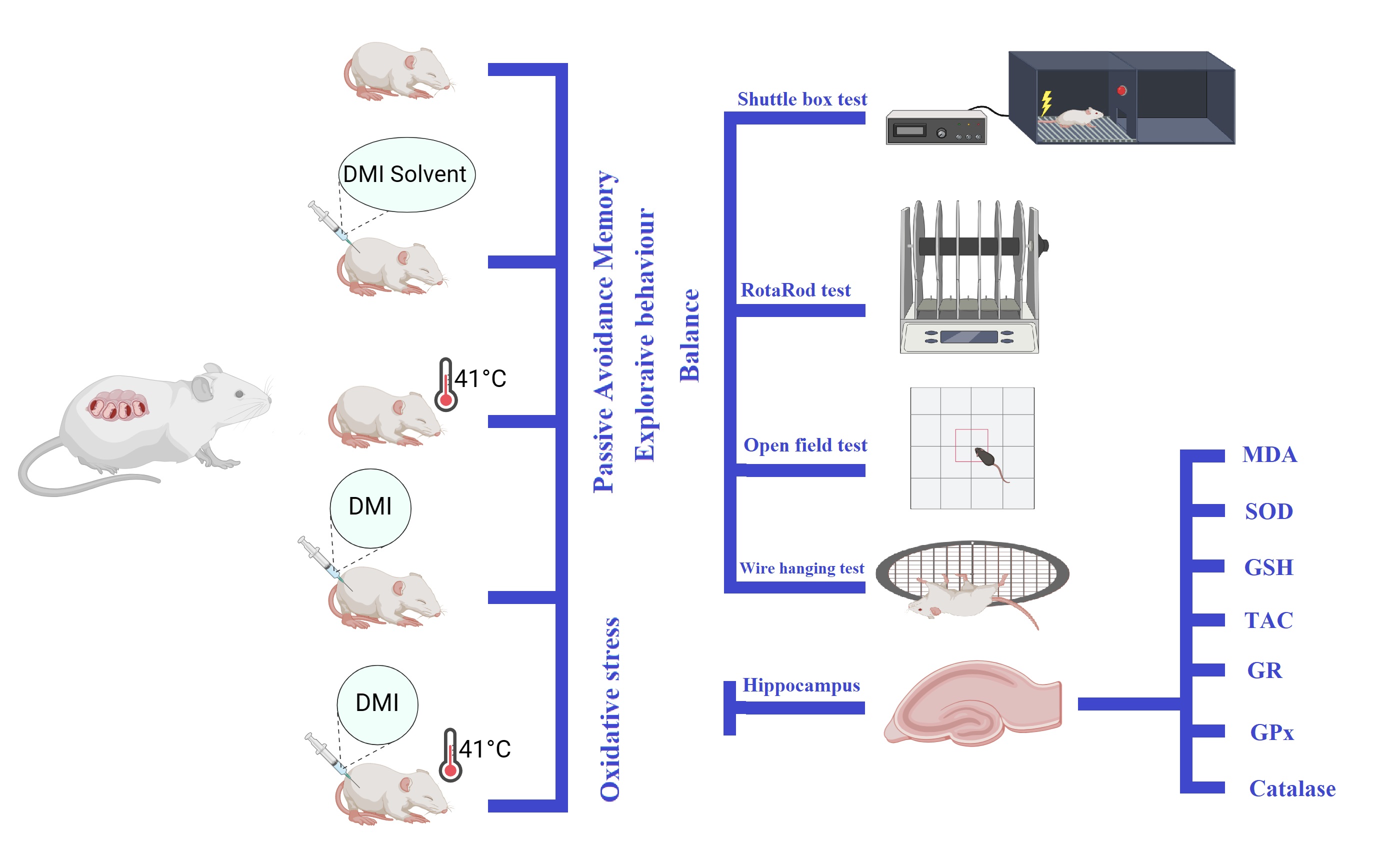

Supplement: S1 File — (JPG) [file pone.0318430.s002.jpg]
